# The effect of competing carnivores on the feeding behaviour of leopards (*Panthera pardus*) in an African savanna

**DOI:** 10.1002/ece3.7608

**Published:** 2021-05-11

**Authors:** Allan Tarugara, Bruce W. Clegg, Edson Gandiwa, Victor K. Muposhi

**Affiliations:** ^1^ Malilangwe Wildlife Reserve Chiredzi Zimbabwe; ^2^ School of Wildlife, Ecology and Conservation Chinhoyi University of Technology Chinhoyi Zimbabwe

**Keywords:** Bait, camera trap, competition, GPS collar, intraguild, kleptoparasitism,predation risk

## Abstract

Knowledge of competition dynamics among Africa’s large carnivores is important for conservation. However, investigating carnivore behaviour in the field can be challenging especially for species that are difficult to access. Methods that enable remote collection of data provide a means of recording natural behaviour and are therefore useful for studying elusive species such as leopards (*Panthera pardus*). Camera traps and Global Positioning System (GPS) collars are powerful tools often used independently to study animal behaviour but where their data are combined, the interpretation of a species’ behaviours is improved. In this study we used data from baited camera trap stations to investigate the feeding habits of leopards at Malilangwe Wildlife Reserve, Zimbabwe. We investigated the influence of spotted hyenas, lions and other competing leopards on the feeding duration of leopards using Generalized Linear Mixed Effects Modelling. To test the influence of competing predators on resting distances from bait sites, eight leopards were fitted with GPS collars. Results showed that leopards spent the shortest time feeding on the baits in the presence of competing male leopards compared to other predators while lion presence caused animals to rest farthest from bait sites. Interaction analysis indicated that small‐bodied leopards spent significantly shorter durations feeding when spotted hyenas were present. Our findings demonstrate that competition from guild carnivores has negative impacts on the food intake of leopards, which may have implications for fitness and survival. This study provides a snapshot of the competition dynamics at bait sites which may give insight to ecosystem level interactions among large carnivores in savanna ecosystems.

## Graphical abstract

Interactions between sympatric carnivores at food sources influence food acquisition and species behaviour. Intraguild competition has fitness, reproductive, and survival implications to subordinate species, and this paper investigates the effect of spotted hyenas, lions, and conspecifics on the feeding habits of leopards in savanna ecosystems.

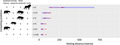

## 1 INTRODUCTION

Competition and predation within the large predator guild are important factors shaping the behaviour and demographics of sympatric carnivores (Caro & Stoner, 2003; Hayward et al., 2006). In the wild, carnivores are known to scavenge on animals that have died of natural causes and to kleptoparasitize kills from each other (Creel, 2001; Höner et al., 2002; Mills & Funston, 2003; Selva et al., 2005). The African leopard (*Panthera pardus*) is a generalist predator (Hayward et al., 2006; Shehzad et al., 2015), actively hunting for its prey but also taking carrion whenever present (Bothma & Walker, 2013; Stuart & Stuart, 1993). Leopards usually cache their kills in bushes and consume them on the ground (Balme et al., 2007; Farhadinia et al., 2020; Karanth & Sunquist, 2000). However, competing guild predators such as spotted hyenas (*Crocuta crocuta*) and lions (*Panthera leo*) often discover and kleptoparasitize cached prey (Domínguez‐Rodrigo, 2001; Pitman et al., 2013; Volmer & Hertler, 2016). Kleptoparasitism negatively affects subordinate predators by reducing their food intake and increasing the risk of injury or death (Bryce et al., 2017; Elbroch et al., 2014; Palomares & Caro, 1999). Feeding in the presence of competing carnivores means leopards must balance food intake and risk (Brown, 1988; Verdolin, 2006). Leopards respond to kleptoparasitism by caching their kills in trees, which reduces interference from non‐climbers such as hyenas, but not from competitors that are capable of climbing such as conspecifics and lions (Balme et al., 2017; Rafiq, 2016).

Investigating intraguild competition in the wild is challenging (Caravaggi et al., 2017). Field observations of animal behaviour enable the recording of responses to various stimuli; however, this has practical challenges where study species occur at low densities or are difficult to access (Caravaggi et al., 2017; Creel, 2001; Vanak & Gompper, 2009). In addition, the presence of human observers in the field may provoke unnatural responses resulting in biased inferences (Caravaggi et al., 2017). Our study presents a novel approach that uses baits and camera traps to create a partially controlled environment where competition among large carnivores can be quantified. To approximate field conditions, we simulated natural feeding sites of leopards by hanging baits in trees at sampling stations which were rigged with camera traps for remote collection of behaviour data.

Our study used data collected from baited camera traps (BCT) and Global Positioning System (GPS) collars to investigate the feeding habits of leopards at Malilangwe Wildlife Reserve, Zimbabwe. A key strength of camera traps is the ability to survey multiple species and where these data are coupled with location information, for example, from GPS collars, a more accurate interpretation of a species’ habits can be achieved (Soisalo & Cavalcanti, 2006). Camera traps also offer the possibility to survey multiple locations simultaneously and are noninvasive thereby causing little disturbance (Tarugara et al., 2019). Two sympatric large carnivores, namely lions and spotted hyenas, and conspecific leopards potentially influence the feeding behaviour of leopards in the study area. Previous studies have demonstrated that competing predators as well as environmental variables influence food intake of carnivores (Brown, 1988; Domínguez‐Rodrigo, 2001; Glen & Dickman, 2005; Périquet et al., 2015; Verdolin, 2006); however, there is little quantitative information on the impact of competing carnivores on leopard feeding behaviour. Our study investigated the effect of lions, spotted hyenas and other leopards on (1) the time leopards spent feeding and (2) the resting distance away from feeding stations. We hypothesized that behavioural responses in leopards are influenced by an individual subject's body size and the species of competitor. The findings of our study may broaden the present knowledge on intraguild competition among large carnivores, possibly influencing policy and management in areas where leopards are a species of conservation interest.

## 2 METHODS

### 2.1 Study area

**FIGURE 1:**
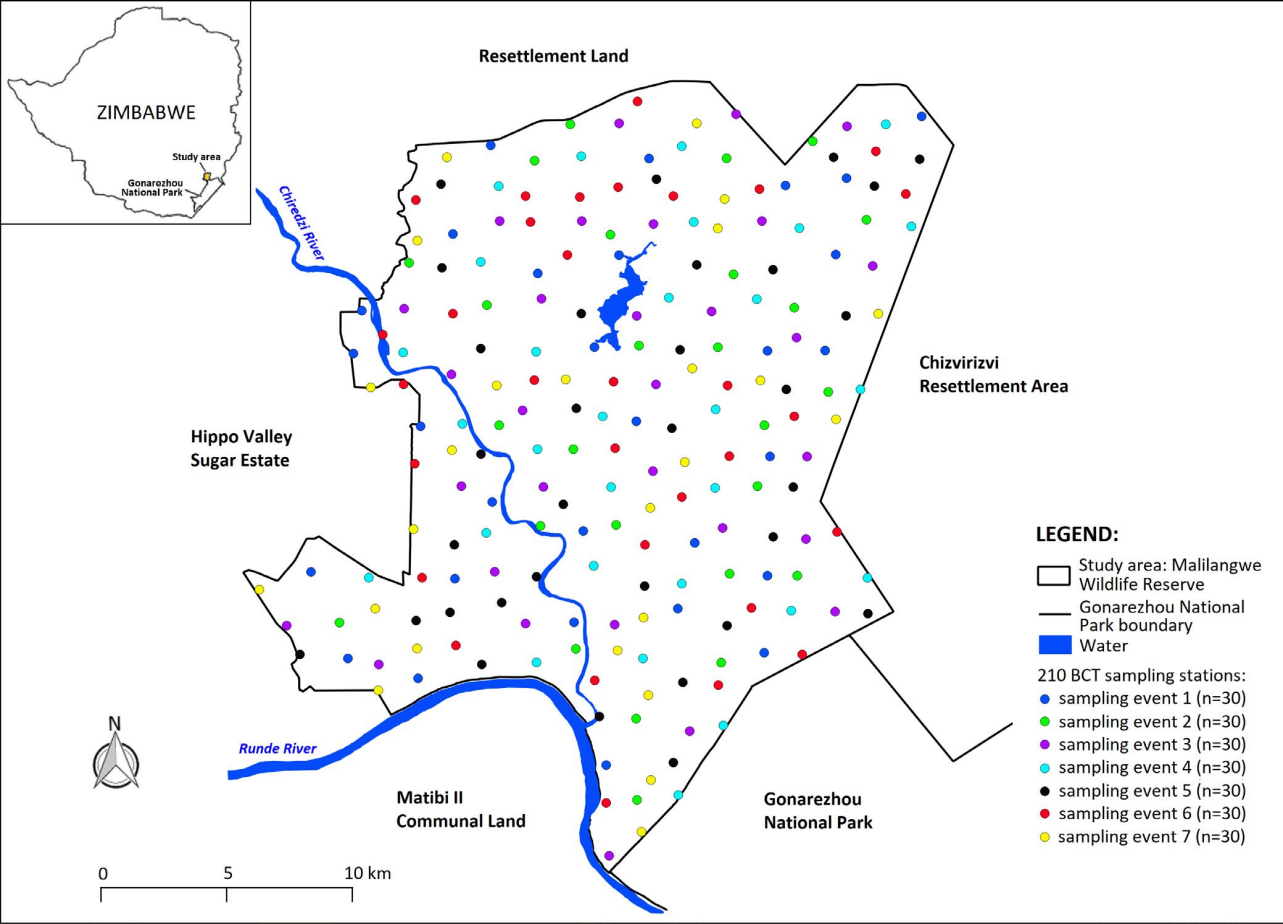
Location of Malilangwe Wildlife Reserve, southeast Zimbabwe. Colored dots represent the location of BCT stations used in the study

The study was conducted on Malilangwe Wildlife Reserve (MWR), a 490‐km^2^ fenced, protected area in the semi‐arid savanna of southeastern Zimbabwe (20°58′ ‐ 21°15′S and 31°47′ − 32°01′E) (Figure 1). MWR is a non hunting property whose main objectives are conservation and community development. Rainfall (mean ≈ 560 mm per annum, *n* = 66 years, coefficient of variation = 34%) is seasonal with approximately 84% of precipitation occurring between November and March. Rainfall patterns are erratic, and the area is prone to droughts. The average minimum and maximum monthly temperatures range from 13.4°C (July) to 23.7°C (December) and 23.2°C (June) to 33.9°C (November), respectively (Clegg & O’Connor, 2017). Altitude ranges from 290 m, in river systems, to 500 m above sea level on sandstone hills (Traill & Bigalke, 2007).

The reserve is generally characterized by open savanna woodland dominated by *Colophospermum mopane*. Vegetation cover is diverse, ranging from grassland to dry deciduous woodland, with 38 vegetation types occurring on soils ranging from 90% sand to 40% clay (Clegg & O’Connor, 2012). The leopard density at MWR is estimated at 0.12 individuals km^−2^ (Tarugara et al., 2019) and the main prey species (density in parentheses) are impala (*Aepyceros melampus*, 13.6 km^−2^), nyala (*Tragelaphus angasii*, 0.38 km^−2^), and bushbuck (*Tragelaphus sylvaticus*, 0.22 km^−2^) (Clegg, 2017). Competing predators include lion (0.1 km^−2^), spotted hyenas (0.12 km^−2^), and wild dog (*Lycaon pictus*, 0.06 km^−2^) (Clegg, 2017).

### 2.2 Collaring of leopards

Between 7 May and 20 June 2017, five adult male and five adult female leopards were captured using walk‐in, fall‐door traps and fitted with Followit GPS/Very High Frequency (VHF) collars (model Tellus Small: Followit, Lindesberg, Sweden). Traps were placed near roads for easy access and in thick scrub for concealment. Impala meat was used as bait and entrails were dragged along the road to the trap to attract passing leopards (see Tarugara et al., 2019). Because leopards are predominantly nocturnal, traps were only operational at night. To minimize stress and injury associated with capture (Sikes et al., 2016), we devised an alarm system that alerted researchers in real time when the trap was tripped. The alarm was rigged by attaching a VHF transmitter to the top rail of the trap, which was switched off using a magnet that was attached to the fall door by a string. When a leopard tripped the setup, the falling door pulled off the magnet which activated the transmitter to send a signal that was picked up by a collaring team which was on standby for each active trap. This was necessary to reduce stress and injury associated with a cat being trapped for a long time (Sikes et al., 2016). Captured leopards were immobilized using a combination of Zoletil and Medetomidine (1.0–0.03 mg/kg body mass), with the anesthetic being darted into the muscular region of the hindquarters. Drug reversal was achieved by subcutaneous injection of Antisedan (at 2.5 mg/mg of Medetomidine) or Yohimbine (at 1 ml/50 kg of body weight). Body measurements of animals were recorded for a separate study (Tarugara et al., 2019b) and following the baited camera trapping protocol described below, a photograph of the right‐side profile of each leopard was taken for identification (Joubert et al., 2020; Tarugara et al., 2019). To protect sedated leopards from lions and hyenas, animals were monitored until they fully recovered from the effects of the anesthesia. All handling procedures were performed by a licensed practitioner (with Zimbabwean Dangerous Drugs License number: 2017/25) following safe and professional guidelines stipulated by the American Society of Mammalogists (Sikes et al., 2016). Ethical clearance for the study was granted by the Chinhoyi University of Technology Ethics Committee (clearance number: 01/17).

### 2.3 GPS collar data collection

Collars were programmed to fix a GPS position at 15‐min intervals from 16:00 to 10:00 and one fix during the hottest part of the day (13:00), when leopards were presumed less active (i.e., 73 positions per day). Collars broadcasted VHF signal Mondays to Fridays from 07:00 to 13:00, and automatic drop‐offs were set at 240 days. From 08 August 2017 to 29 January 2018, collared individuals were tracked using VHF telemetry and GPS data were remotely downloaded onto a laptop computer via a Tellus RCD‐04 Ultra High Frequency terminal (Followit, Lindesberg, Sweden).

### 2.4 Camera trap data collection

From 1 July to 22 October 2017, photographic data for a population survey were collected over 210 BCT stations, distributed across the study area using a stratified random sampling strategy (see Tarugara et al., 2019). The study area was surveyed over seven separate sampling events, each comprising 30 trapping stations and lasting 14 days (i.e., a total survey effort of 98 sampling days) (Figure 1). At each sampling station, two trees were chosen, one for the bait and the other for the camera. Impala carcasses, harvested by licensed professional hunters in accordance with Zimbabwean regulations and ethics (Government of Zimbabwe, 1975; Lewis et al., 1997), were used as bait. MWR has a healthy impala population (density =13.6 km^−2^) (Clegg, 2017) and the number harvested for baits was within the approved annual management quota of 300 (Zimbabwe Parks and Wildlife Management quota allocation, 2017). At each sampling station, a carcass was secured to the bait‐tree and a leading pole was placed against the tree to provide easy access for leopards (Tarugara et al., 2019). A Cuddeback C2 infrared camera (Cuddeback, WI, USA) was positioned on the camera‐tree to the right of each bait, with its line of sight at 90° to the leading pole. This way, when a leopard climbed up the pole to feed, a photograph of the right profile of the animal was taken (see Joubert et al., 2020 for more details). The trigger delay between photographs was set to 1 min, and the clock was adjusted to match the default Greenwich Meridian Time fixes from the collars.

### 2.5 Data analysis

Leopards were identified from photographs using unique spot patterns on their right flanks (Tarugara et al., 2019). GPS fixes were obtained from 8 (four males and four females) out of the 10 collared leopards. A male leopard shrugged off its collar and one female left the property early in the study. We filtered the dataset to include only GPS fixes for the period when the BCT stations were active (all collars were functional over this period) and these data were imported into Quantum GIS (QGIS) v2.18 (QGIS Development Team, 2016) for analysis. Leopards have a keen sense of smell and use this to locate carrion and detect chemical secretions of other animals (Law et al., 1997; Press & Minta, 2000). It was assumed that the smell of a carcass could be broadcast by air movements and alert leopards that were traveling a distance away to the presence of a bait. To determine the effective detection radius, defined as the distance around the bait within which a roaming leopard would smell the presence of a bait, approach distances were measured from the closest GPS fix to the bait in QGIS and “hits” or “walk past” events recorded. Since leopards could approach a bait site from any direction, a radius was created around each bait by assigning the average value of all approach distances that resulted in “hits.” If GPS points from a roaming leopard came within the effective detection radius, a bait was considered to have been discovered. The presence of spotted hyenas or lions at a bait site may have influenced the outcome post detection as leopards could avoid approaching sites where competitors were already present. For this reason, records showing competing predators at bait sites on initial approaches were not included in this analysis to eliminate bias. It was also recorded if a leopard detected a bait but chose not to feed, that is, if a GPS fix was within effective detection radius but the leopard was not photographed at the bait.

Where a leopard had “hit” a bait, the time spent feeding in the presence or absence of competitors was recorded. A feeding event was defined as an unbroken time record where a leopard was photographed on a bait. Camera traps were programmed to take photographs at one‐minute intervals, and therefore, the time spent feeding in minutes was calculated by counting the number of photographs taken of the leopard at the bait in an unbroken time sequence. Feeding events were categorized according to the presence or absence of competing carnivores in the photographs. In general, feeding events in the presence of competitors were short (only a few minutes), and in most cases, assessment was straightforward because the competitor was often present in the photographs in an unbroken sequence up until the moment the leopard left the bait. However, because the camera's field of view was such that a competitor could be present but be outside the photographic frame, each feeding event was investigated in its own objective way and logical deductions made. For example, if a competitor was visible in a photograph, missing in the next one and present again in the next, then the competitor was deemed not to have left the bait site even though it was not visible in the middle photograph because one minute was insufficient time for the competitor to have traveled sufficiently far away for its presence not to have an influence. Records of both collared and uncollared leopards were used for feeding time analysis. The presence or absence of testes was used to distinguish the sex of competing leopards (Tarugara et al., 2019). Baits were also accessible to lions because they can climb trees. Lions robbed baits that they discovered. As such, there was exploitation competition with lions and leopards and interference competition with spotted hyenas (no baits were removed by hyenas).

Between feeding events, a collared leopard could leave the bait to rest a distance away. A waiting site was defined as a cluster of ≥3 consecutive GPS positions that was within 1 000 m of a bait and from which a leopard returned to the bait site. A cut‐off of 1 000 m was chosen because within this distance it was assumed that a leopard could monitor activity around a bait and assess risk. If a leopard rested beyond 1 000 m it was assumed to have lost interest in the bait. A leopard could wait at multiple sites between feeding events, and therefore, the distance in meters was measured from the bait to each waiting site and the mean distance calculated for each cluster of GPS points representing a waiting animal. The number of days a leopard took to return to a previously discovered bait was also calculated.

We used generalized linear mixed models (GLMMs) to analyze the effect of competing predators on the behaviour of leopards at bait stations (Bates et al., 2014; R Core Team, 2017). *Time spent feeding* and *waiting distance* were used as dependent variables while *body size* (small, medium, or large), *spotted hyenas* (presence or absence)*, competing female leopard* (presence or absence)*, competing male leopard* (presence or absence) and *lion* (presence or absence) were used as fixed effects. Multiple records from the same leopard or from the same baiting station were not independent; therefore, we used *sampling station ID* (spatial non‐independence) and *leopard ID* (within‐subject non‐independence) as random effects and ran models in the GLMM as,

$$\text{Feeding time}∼body\;size+spotted\;hyenas+competing\;female\;leopard+competing\;male\;leopard+lion+\;\left(1|sampling\;station\;ID\right)\;+\;\left(1|leopardID\right)\;\text{an}$$

$$\text{Waiting distance}\;∼spotted\;hyenas+competing\;female\;leopard+competing\;male\;leopard+lion+\;\left(1|sampling\;station\;ID\right)\;+\;\left(1|leopardID\right)$$

Because waiting distance analysis was limited to the eight collared leopards, sample sizes for body size categories were insufficient, and therefore, this effect was dropped from the model. The dependent variables were not normally distributed within each combination of fixed effects and the data were right‐skewed. Consequently, using the loglink function, the model for feeding time was specified using a Poisson distribution while a Gamma distribution was used for the waiting distance model. The *emmeans* package of R (Lenth et al., 2018) was used to calculate the estimated marginal means (and 95% confidence intervals) for the various combinations of fixed effects. Comparisons, adjusted using the Tukey method, were displayed graphically to show significant differences (α = 0.05) between the estimated marginal means.

The body size of a feeding leopard can influence its ability to stand‐off competition (Skinner & Smithers, 1990) and consequently determine feeding time. A study by Tarugara et al. (2019b) showed that the body length of leopards can be accurately estimated from measurements performed on camera trap photographs (mean error  ​= 2.0 cm). We used body length as a proxy for size (Tarugara et al., 2019b) and categorized it into three classes, small (body length <70 cm), medium (≥70 cm – 79 cm), and large (body length ≥80 cm) bodied animals. The interaction of body size and competing predator species was tested using GLMMs for combinations with sufficient sample sizes (*n* ≥ 10) as,

$$\text{Feeding time}\;∼body\;size+competing\;predator+body\;size\;*\;competing\;predator+\;\left(1|sampling\;station\;ID\right)\;+\;\left(1|leopardID\right)$$

Additive models for combinations of body size and each competing predator were compared with the respective interaction model using AICs (R Core Team, 2017).

## 3 RESULTS

**FIGURE 2:**
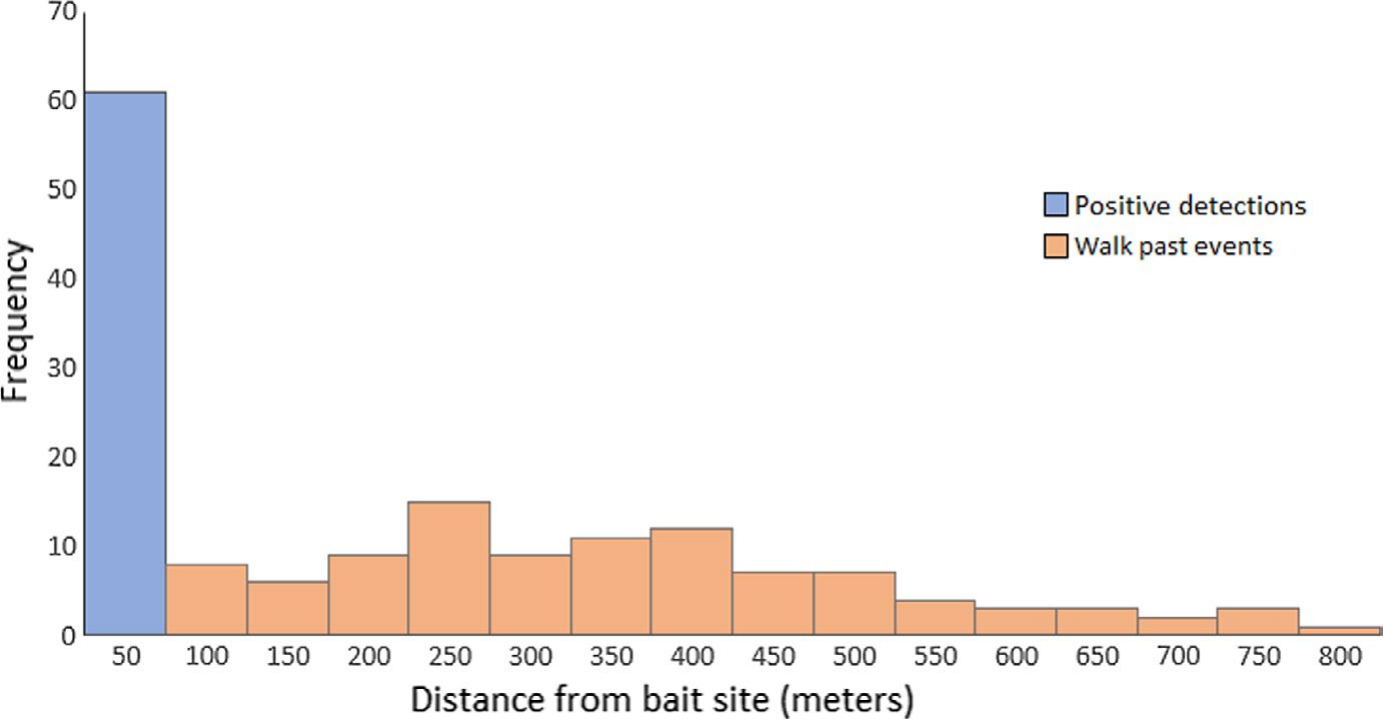
Frequency (*n*=count data) of bait detection and misses by roaming study leopards with distance from sampling stations. Detected baits were subsequently visited by leopards

All positive detections of baits were within 50 m of the site with distances beyond this threshold resulting in “walk past” events (Figure 2). Spotted hyenas detected baits earliest (mean =2.7 ± 2.2 days, *n* = 183 hits), followed by leopards (collared and uncollared, 5.5 ± 3.6 days, *n* = 179 hits) and lions (6.6 ± 4 days, *n* = 59 hits), respectively. Over the duration of the sampling period, collared leopards discovered 75 of the 210 baits, with 8 of these not being fed on. In addition, collared leopards discovered an average of 1.9 baits (range =1–4) per sampling event and when displaced (*n* = 32 observations [spotted hyenas =15, male leopards =9, female leopards =3, lions =5]), they revisited bait sites after an average of 4.3 ± 2.3 days. The global dataset contained records of multiple leopards photographed at the same bait site (same day: *n* = 63, same sampling event: *n* = 55). The dataset for time spent feeding on a bait contained 325 events from 16 leopards recorded at 68 camera stations, while that for waiting distance was made up of 239 events from eight collared leopards at 59 stations.

### 3.1 The effect of competing carnivores on feeding time of leopards

The GLMM of time spent feeding at sampling stations showed that spotted hyenas, male leopards, and lions reduced the time spent feeding by leopards at bait sites. The presence of competing male leopards resulted in the greatest reduction in feeding time followed by lions and spotted hyenas, respectively (Table 1). A feeding leopard's body size and competition from female leopards did not influence feeding time.

**TABLE 1:** GLMM results of the model: Time spent feeding ~*body size* + *spotted* *hyenas* + *competing* *female leopard* + *competing* *male leopard* + *lion* + (1|*sampling station ID*) + (1| *leopard* *ID*). Fixed effects with Pr (>|z|) values <0.05 were considered significant

A. Fixed effects	Estimate	Std. Error	*z* value	Pr (>|z|)
Intercept	2.59	0.47	5.53	3e−08
Body size (medium)	−0.94	0.61	−1.53	0.127
Body size (small)	−0.10	0.64	−0.16	0.874
Spotted hyenas	−0.74	0.07	−10.92	2e−16
Competing female leopard	−0.22	0.13	−1.69	0.091
Competing male leopard	−1.79	0.23	−7.64	2e−14
Lion	−1.74	0.37	−4.74	2e−06

B. Random effects		Variance		Std. Dev
Station ID		0.41		0.64
Leopard ID		0.73		0.85

**FIGURE 3:**
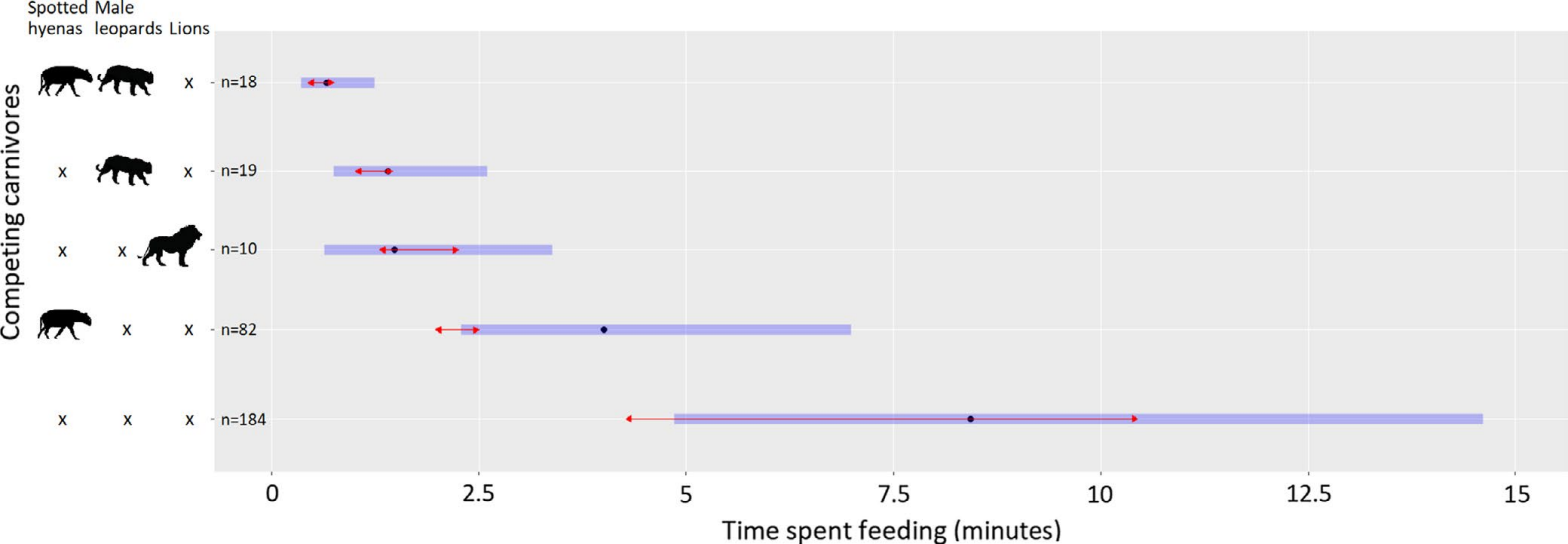
Effect of competing predators on the time spent feeding on baits by collared leopards. *n* represents sample size and black dots denote estimated marginal means. Blue bars and red arrows represent 95% confidence intervals and results of pairwise comparisons between marginal means, respectively. Categories with overlapping arrows were not significantly different (*p* > 0.05)

The marginal means showed that the effect of a competing male leopard in combination with spotted hyenas resulted in the greatest reduction in feeding time (Figure 3). Pairwise comparisons of marginal means showed that the effects of competing male leopards, lions, and spotted hyenas on feeding time were not significantly different from each other (Figure 3).

Only the spotted hyenas and body size combinations had sufficient sample sizes for interaction analysis and model comparison between the additive and interactive models using AICs showed that the interaction model better explained the variance in feeding time (Table 2). The interaction effect of small‐bodied leopards and spotted hyenas was significant indicating that small leopards spent a shorter time feeding when spotted hyenas were present (Table 3).

**TABLE 2:** Results of body size and spotted hyenas model ranking using the Akaike information criterion (AIC)

Model	Df.	AIC
Feeding time ~*body* *size* + spotted *hyenas* + (1|*Sampling station ID*) + (1|*Leopard ID*)	6	2,820.51
Feeding time ~*body* *size* + *spotted* *hyenas* + *body* size * spotted hyenas + (1|*Sampling station ID*) + (1|*Leopard ID*)	8	2,809.19

**TABLE 3:** GLMM results of the model: Time spent feeding ~ body *size* + *competing* *hyenas* + *body* *size* * *spotted hyenas* + (1|*sampling station ID*) + (1|*leopard* *ID*). *n* denotes sample size and Pr (>|z|) values <0.05 were considered significant

A. Fixed effects	*n*	Estimate	Std. Error	z value	Pr(>|z|)
Intercept		2.19	0.37	5.91	3e−09
Body size (medium)	139	−1.11	0.52	−2.12	0.034
Body size (small)	88	−0.55	0.53	−1.04	0.297
Spotted hyenas’ presence	82	−0.52	0.11	−4.56	5e−06
Body size (medium): spotted hyenas’ presence	36	−0.25	0.15	−1.67	0.094
Body size (small): spotted hyenas’ presence	18	−0.83	0.22	−3.81	0.000

B. Random effects			Variance		Std. Dev
Station ID	68		0.44		0.66
Leopard ID	16		0.45		0.67

### 3.2 Response of waiting distance to presence of other carnivores

The presence of competing predators had a significant influence on the distance feeding leopards waited from bait sites (Table 4). Lions had the strongest influence on waiting distance compared to other competitors.

**TABLE 4:** GLMM results of the model: Waiting distance ~ *spotted* *hyenas* + *competing* *female leopard* + *competing* *male leopard* + *lion* + (1|*sampling station ID*) + (1|*leopard* *ID*). Fixed effects with Pr (>|z|) values <0.05 were considered significant

A. Fixed effects	Estimate	Std. Error	*t* value	Pr (>|z|)
Intercept	3.50	0.25	14.23	2e−16
Spotted hyenas	0.00	0.16	0.03	0.979
Competing female leopard	−0.01	0.30	−0.04	0.970
Competing male leopard	0.77	0.30	2.59	0.010
Lion	2.18	0.47	4.60	4e−06

B. Random effects		Variance		Std. Dev
Station ID		0.42		0.65
Leopard ID		0.34		0.58
Residual		0.86		0.93

**FIGURE 4:**
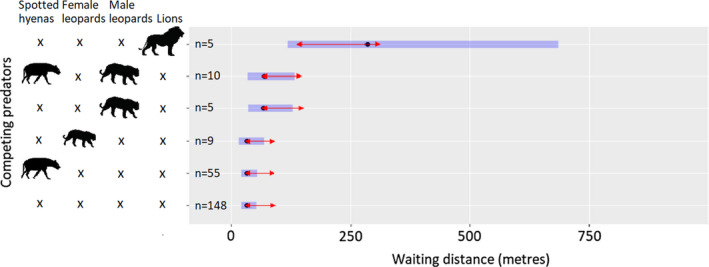
The effect of competing predators on the waiting distance of collared leopards from baits. *n* represents sample size, and black dots denote estimated marginal means. Blue bars and red arrows represent 95% confidence intervals and the results of pairwise comparisons between marginal means, respectively. Categories with overlapping arrows were not significantly different (*p* > 0.05)

The presence of lions was associated with longer waiting distances from bait sites when compared to other competitors (lions =292 m, 95% CI [108 – 794 m, *n* = 5], competing male leopards =71 m, 95% CI [35 – 147 m, *n* = 5], spotted hyenas =33 m, 95% CI [20 – 56 m, *n* = 55]) (Figure 4).

## 4 DISCUSSION

Kills and natural mortalities of prey animals may represent points of inter‐ and intraspecific carnivore conflict (Durant, 2000; Toïgo & Gaillard, 2003). Where the density of carnivores is high, competition killing can be common (Caro & Stoner, 2003) either for food or elimination of an ecological competitor (Ritchie & Johnson, 2009). Leopards often climb trees to escape larger guild competitors as encounter can have costs such as mortality and loss of kills (Bailey, 2005; Balme & Hunter, 2013; Lourenço et al., 2014).

Our study demonstrated that baits can be used to provide novel insights into the competition dynamics of co‐existing species. Baits allowed researchers to simulate feeding stations of leopards under field conditions that could be monitored by cameras. Without baits, it would be difficult to find sufficient natural kills to make robust inferences and even if this were possible, it would be difficult to observe the interactions at the kills without interfering with the behaviour of leopards and their competitors. However, it should be noted that use of baits is not an exact simulation of natural kills because the baits were not the leopards’ own kills, and this might have made them more cautious (i.e., feeding leopards were essentially scavenging a kill).

### 4.1 The influence of competing predators on the feeding behaviour of leopards

The presence of spotted hyenas, lions, and competing male leopards had a negative influence on the time leopards spent feeding on baits. Of all the competing predators, male leopards had the strongest influence, with feeding animals spending the shortest time feeding on baits in their presence. The ability of competing leopards to skilfully climb the leading pole or trees means that they could rapidly access the bait, thereby increasing their level of threat relative to the other species. Male leopards pose more threat to feeding animals than female leopards due to their larger body size and competitive dominance (Skinner & Smithers, 1990). Hyenas cannot climb, and while lions can climb trees, they are not particularly skilled at it, which may have lowered their perceived threat. A study in Phinda Game Reserve, South Africa, showed that intraspecific killing in leopards had a stronger effect than interspecific conflict (Balme et al., 2009). Similarly, Balme and Hunter (2013) reported that male leopards were responsible for most infanticide deaths in Sabi Sand Game Reserve, South Africa. Evidence at MWR also supports this because from 1998 to 2018, seven leopard deaths were recorded from conspecific males and only three deaths from lions.

Because our analysis of feeding time considered unbroken feeding records, this meant breaks between feeding sessions resulted in multiple feeding events with short feeding times, even in the absence of competitors. This had the net effect of depicting a small difference between contested and uncontested feeding times. However, the distinction is actually greater than recorded because leopards in uncontested situations can alternately break and start feeding thereby resulting in longer cumulative feeding times. Our findings showed that interaction competition between small‐bodied leopards and spotted hyenas was significant. Small‐bodied leopards spent a shorter time feeding when spotted hyenas were present. This may probably be because small‐sized leopards felt threatened by spotted hyenas and therefore minimized food intake where displacement may be imminent.

Of all predators, lions caused collared leopards to wait farthest from bait sites. In the absence of lions, leopards in our study generally stayed close to baits (<72 m). This indicates that when choosing resting locations, leopards considered lions to be the greatest threat. Leopards are competitively subordinate to lions; they often avoid contact or conflict (Bischof et al., 2014). Longer waiting distances were observed in the presence of lions possibly because when resting on the ground, leopards perceived lions to be a greater threat than the other competitors. In our study, sample sizes for competing male leopards and lions’ presence were small for waiting distance analysis because the data were based on GPS collar fixes and therefore inferences were limited to eight collared individuals only. Collaring a large proportion of the population may have improved the resolution of the data but resources for our study were limited. The low sample sizes for some of the treatment effects mean that the results for these effects should be treated with caution.

Feeding in the presence of competing predators is inherently risky, and the trade‐offs between time spent feeding and safety have been previously studied (Brown, 1988; Verdolin, 2006). Energetic costs and safety at feeding sites are important determinants for the survival, reproduction, and overall fitness of carnivore populations (Durant, 2000; Caro & Stoner, 2003; Hunter et al., 2007; Watts & Holekamp, 2008; Rafiq, 2016; du Preez et al., 2017). If carrion can be easily obtained, leopards readily scavenge even where live prey is abundant (Gonzalez & Piña, 2002). Scavenging can be crucial to the survival of young, old, and handicapped individuals with limited hunting proficiency (Bailey, 1993; Bauer et al., 2005; Focardi et al., 2017). Leopards sometimes consume kills on the ground but where intraguild competition for kills or carrion is high, they usually hoist their prey up trees to avoid being kleptoparasitized (Balme et al., 2017; Stein et al., 2015). Because lions do not balance well on the leading pole, we believe that the baits used in our study closely simulated feeding sites of leopards in the wild and that the observations closely reflect the species’ natural behaviour at feeding sites.

### 4.2 Implications

Our study showed that male leopards at MWR can significantly influence the feeding duration of other leopards at feeding locations. Intraspecific hostility can occur within and between sexes and among leopards of all ages (Farhadinia et al., 2018) and is associated with competition at kills (Steyn & Funston, 2006) or over territory (Balme & Hunter, 2004). Our findings of a strong competitive effect from male leopards were similar to a study by Balme et al. (2017) which showed that male leopards were responsible for 88% of intraspecific kleptoparasitism at Sabi Sand Game Reserve, South Africa. In the wild, antagonistic encounters between unfamiliar individuals occur (White & Harris, 1994) and the level of risk increases with increasing density, with associated competition for space, mates, and food (Glen & Dickman, 2005). Intraspecific killing accounts for 43% of leopard deaths in protected areas of South Africa (Swanepoel et al., 2015) and young adults and females are most vulnerable. This may have implications on the reproductive ecology and fitness of leopard populations. Where leopards are sport‐hunted, adult male animals are selectively taken out. Removing males may result in an increase in population growth in a manner akin to mesopredator release (Courchamp et al., 1999; Crooks & Soulé, 1999).

In our study, spotted hyenas and leopards discovered most of the baits (87% and 85% respectively) while lions found fewer (28%). This indicates that spotted hyenas and leopards may have more effective olfactory capabilities for detecting carrion than lions although this may have been influenced by the densities and ranging behaviour of each species (Mills, 1989). Despite the strong effect lions have on feeding and waiting behaviour, their overall influence on leopard populations might not be as marked as that of spotted hyenas because they did not find baits easily. It should, however, be noted that under natural situations actual kills are often associated with vocalizations, which may increase the probability of detection by competitors (Jones et al., 2015). In addition, bias was introduced by placing baits up trees and since leopards sometimes consume kills on the ground (Balme et al., 2017; Bothma, 1998), this may have masked the true competitive effect of spotted hyenas. Spotted hyenas have been reported to exert a strong competitive influence on other guild members (Volmer & Hertler, 2016), and they are the main carnivore species impacting the feeding ecology of leopards at Sabi Sand and Selati Game Reserves, South Africa (Balme et al., 2017; Comley et al., 2020). However, where lion densities are high, negative impacts on the survival or persistence of leopard populations may be apparent, either through competition for food or direct killing of individuals or their young (Donadio & Buskirk, 2006; du Preez et al., 2015).

Predation and feeding success are facilitated by physical features of the habitat (Davidson et al., 2012; Davies et al., 2016; Hopcraft et al., 2005; Kauffman et al., 2007). The presence of tall trees is an important factor in the feeding ecology of leopards because they hoist kills up trees in an attempt to avoid kleptoparasitism (Balme et al., 2017). For example, spotted hyenas were present at 82% of the baits at the same time leopards were feeding and such a high rate of interference competition may necessitate the habitual hoisting of prey by leopards as a behavioural response. The effect of interactions observed at the scale of the bait sites in our study may also exist at the ecosystem level. For example, competing predators may influence the ranging behaviour of leopards (Dröge et al., 2017; Miller et al., 2018; Odden et al., 2010). This could be a focus of future research.

## 5 CONCLUSION

Using a novel experimental design, our study has demonstrated that the effect of competing predators on the feeding behaviour of leopards can be successfully quantified by simulating field attributes of feeding sites at sampling stations. The findings confirmed our hypothesis that the type of competitor species present at feeding stations influences behavioural responses in leopards. We conclude that competing male leopards and lions can negatively impact feeding times and resting distances of leopards and this may have fitness and survival implications. We recommend that researchers take advantage of baited camera trapping surveys to widen the scope of research by collecting behavioural data for leopards and similar species.

## CONFLICT OF INTEREST

The authors declare no conflict of interest.

## AUTHORS’ CONTRIBUTIONS

- Allan Tarugara conceived and designed the experiments, performed the experiments, analyzed the data, contributed reagents/materials/analysis tools, prepared figures and/or tables, authored or reviewed drafts of the paper, and approved the final draft.
- Bruce W. Clegg conceived and designed the experiments, performed the experiments, analyzed the data, contributed reagents/materials/analysis tools, authored or reviewed drafts of the paper, and approved the final draft.
- Edson Gandiwa and Victor K. Muposhi authored or reviewed drafts of the paper, and approved the final draft.

## Data Availability

Data for this study are available from the Dryad Digital Repository as: Tarugara, Allan; Clegg, Bruce; Gandiwa, Edson; Muposhi, Victor (2021), The effect of competing carnivores on the feeding behavior of leopards (Panthera pardus) in an African savanna, https://doi.org/10.5061/dryad.1rn8pk0qx
